# A novel algorithm based on bi-random walks to identify disease-related lncRNAs

**DOI:** 10.1186/s12859-019-3128-3

**Published:** 2019-11-25

**Authors:** Jialu Hu, Yiqun Gao, Jing Li, Yan Zheng, Jingru Wang, Xuequn Shang

**Affiliations:** 1 School of Computer Science, Northwestern Polytechnical University, Xi’an, 710072 China; 2 Centre for Multidisciplinary Convergence Computing, School of Computer Science, Northwestern Polytechnical University, Xi’an, 710129 China; 3 Ming De College, Northwestern Polytechnical University, Xi’an, 710124 China

**Keywords:** LncRNA-disease association, Bi-random walks, Gene ontology, Interaction profile

## Abstract

**Backgrounds:**

There is evidence to suggest that lncRNAs are associated with distinct and diverse biological processes. The dysfunction or mutation of lncRNAs are implicated in a wide range of diseases. An accurate computational model can benefit the diagnosis of diseases and help us to gain a better understanding of the molecular mechanism. Although many related algorithms have been proposed, there is still much room to improve the accuracy of the algorithm.

**Results:**

We developed a novel algorithm, BiWalkLDA, to predict disease-related lncRNAs in three real datasets, which have 528 lncRNAs, 545 diseases and 1216 interactions in total. To compare performance with other algorithms, the leave-one-out validation test was performed for BiWalkLDA and three other existing algorithms, SIMCLDA, LDAP and LRLSLDA. Additional tests were carefully designed to analyze the parameter effects such as *α*, *β*, *l* and *r*, which could help user to select the best choice of these parameters in their own application. In a case study of prostate cancer, eight out of the top-ten disease-related lncRNAs reported by BiWalkLDA were previously confirmed in literatures.

**Conclusions:**

In this paper, we develop an algorithm, BiWalkLDA, to predict lncRNA-disease association by using bi-random walks. It constructs a lncRNA-disease network by integrating interaction profile and gene ontology information. Solving cold-start problem by using neighbors’ interaction profile information. Then, bi-random walks was applied to three real biological datasets. Results show that our method outperforms other algorithms in predicting lncRNA-disease association in terms of both accuracy and specificity.

**Availability:**

https://github.com/screamer/BiwalkLDA

## Background

It suggests that only 1.5% of genes in the human genome were protein-coding genes, which are twice as many as that of worm and fruit fly [1]. However, 74.7% of the human genome is involved in the process of primary transcripts [2]. It implies that non-coding RNAs play major roles in the regulation of gene expression. The presence or absence of some non-coding RNAs could down- or up-regulate a cascade of gene expression, which could be drug targets for medical therapy of a disease. Many researchers put efforts in to the discovery of the long non-coding RNAs function. Recent studies have found strong association between lncRNA and diseases. It shows that many lncRNAs play as some functional roles in diverse biological processes, such as cell proliferation, RNA binding complexes, immune surveillance, neuronal processes, morphogenesis and gametogenesis [3]. Their dysfunction may cause various diseases. For example, HOTAIR would induce androgen-independent (AR) activation, which plays a central role in establishing an oncogenic cascade that drives prostate cancer progression. It is also a causal reason for AR-mediated transcription programs in the absence of androgen [4]. Therefore, the prediction of lncRNA function would give us a new way to understand the regulation mechanism and disease pathology. There is an urgent demand for the development of fast and accurate algorithm to predict lncRNA-disease association.

Many computational tools have recently been developed to predict potential lncRNA-disease association and functional patters in biological networks [5–10]. Functional patterns in biological networks. These computational methods are majorly in three categories. One of them is based on the idea of matrix factorization. Matrix factorization can be seen as a linear model of latent factors. In these methods, a corresponding latent factor is generated for each lncRNA and disease. Then, it uses a dot product of the latent factors to represent their similarity. The objective function of matrix factorization is to learn the optimal latent factors which can minimize the prediction error. Recently, these methods have been widely used in the prediction of lncRNA-disease relationship. For example, MFLDA reduces the high dimension of heterogeneous data sources into low-rank matrices via matrix tri-factorization, which can help to explore and exploit their intrinsic and shared structure [11]. SIMCLDA translates the lncRNA-disease association prediction problem into a recommendation, which can be solved with inductive matrix completion (IMC) [12]. However, matrix factorization may also bear the risk of over-fitting and the problem of costing-time complexity. Another type of methods is based on the idea of "guilt-by-associate". They are intuitively guided by the assumption that similar disease or lncRNA have similar connection patterns. If disease (A) and lncRNA (A) are known to be related, and disease (A) and disease (B) are very similar. We can infer disease (B) may also related to lncRNA (A). Obviously, the performance of these algorithms heavily depends on the accuracy of the similarity measures. Many "guilt-by-association" algorithms have been proposed. For example, RWRlncD infers potential human lncRNA-disease associations by implementing the random walk with restart method on a lncRNA functional similarity network [13]. IRWRLDA predicts novel lncRNA-disease associations by integrating known lncRNA-disease associations, disease semantic similarity, and various lncRNA similarity measures and make prediction based on improved Random Walk with Restart [14]. The third type of methods focus on classification. Feature extraction was performed on the complex network. Binary classifiers could be applied in the following step to predict whether there exists a connection between lncRNAs and diseases. Another typical prediction algorithm is LRLSLDA, which constructs a cost function in lncRNA and disease space and makes prediction by combining several classifiers in the lncRNA and disease space into a single classifier [15]. LDAP predicts potential lncRNA-disease associations by using a bagging SVM classifier based on lncRNA similarity and disease similarity [16].

In this paper, we proposed a novel algorithm, BiWalkLDA, to predict potential lncRNA-disease associations. The design of BiwalkLDA was intuitivly guided by the assumption of "guilt-by-associate". In order to construct more accurate similarity network, we integrate two types of data from interaction profiles and gene ontology. Furthermore, our method was designed to solve the cold-start problem. BiWalkLDA uses bi-random walks algorithm to predict lncRNA-disease association base on a similarity network we constructed. The experiments were carried out on three real datasets downloaded from the LncRNADisease database [17]. Algorithm performance were evaluated by using Leave-one-out cross validation (LOOCV). Results show that BiWalkLDA outperforms other four state-of-art algorithms, meanwhile it is robust on different datasets and parameters in predicting novel lncRNA-disease associations.

## Methods

### Construction of disease similarity networks

Association patterns were commonly used to calculate disease similarity [14, 18]. In that case, disease similarity will depend on known LncRNA and disease association. Because of the lack of these prior knowledge in lncRNA-disease association, we considered to use gene ontology as an additional information. Gene ontology informations are obtained from previous work [12], which downloaded association between genes and gene ontology terms of human being from Ensemble database [19] and derived disease-gene associations from DisGeNet database [20]. For each disease, we can get the corresponding GO set. Then we use jaccard similarity to measure the similarity between the two sets. The calculation process is shown in the following formula:

$$S_{GO}(d_{i}, d_{j}) = \frac{|{GO}_{d_{i}}\cap {GO}_{d_{j}}|}{|{GO}_{d_{i}}\cup {GO}_{d_{j}}|}$$

where ${GO}_{d_{i}}$ and ${GO}_{d_{j}}$ are two sets of gene ontology terms of disease *d*_*i*_ and *d*_*j*_, respectively. Like previous algorithms, we also construct disease similarity networks by using known disease and LncRNA associations. The construction process can be divided into two steps: (1) construction of an adjacency matrix $A_{n_{l} \times n_{d}}$, where *n*_*l*_ is the number of lncRNA and *n*_*d*_ is the number of diseases. *A*_*ij*_=1 represent that the *i*^*th*^ lncRNA is associated with *d*_*j*_, otherwise *A*_*ij*_=0. (2) With the matrix *A*, we referred *IP*(*d*(*i*)) to the *i*^*th*^ column of *A*, which is the interaction profile of disease *d*_*i*_. IP(d(i)) is a binary vector of length *n*_*l*_ and represents an association pattern of disease d(i). Then we calculate the similarity between two diseases based on the gaussian linear kernel,

$$S_{GKD}(d_{i}, d_{j}) = \exp(-\gamma_{d}||IP(d(i))-IP(d(j))||^{2})$$

where −*γ*_*d*_ is the bandwidth of kernel which is calculated as follow:

$$\gamma_{d} = 1/\left(\frac{1}{n_{d}}\sum_{i=1}^{n_{d}}{||IP(d(i))||^{2}}\right)$$

Here *n*_*d*_ is the number of diseases. Up to now, we have constructed *S*_*GKD*_ based on known association between lncRNA and disease and *S*_*GO*_ based on disease-related GO set. Then we use a simple linear model to fuse the two similarity networks.

$$S_{d} = \alpha S_{GO} + (1-\alpha)S_{GKD}$$

Here *α* is a hyperparameter that control the proportion of *S*_*GKD*_ and *S*_*GO*_. If *α*=1, disease similarity only be calculated base on gene ontology information. If *α*=0, disease similarity only be calculated base on known disease-lncRNA associations. When the matrix is sparse, it would be better to give a large *α* so that similarity rewards can be obtained from geneontology. This technique makes the algorithm more robust

### Construction of lncRNA similarity network

Similar to the previous process, we calculate lncRNA gaussian similarity based on known disease-lncRNA association. First, we use *IP*(*l*(*i*)) which is the *i*^*th*^ row of *A* to represent the interaction profile of lncRNA *l*(*i*). *IP*(*l*(*i*)) is a binary vector of length *n*_*d*_ and represents an association pattern of lncRNA *l*(*i*). Then lncRNA gaussian similarity was calculated base on the following formula:

$$S_{GKL}(l_{i}, l_{j}) = \exp\left(-\gamma_{l}||IP(l(i))-IP(l(j))||^{2}\right)$$

$$\gamma_{l} = 1/\left(\frac{1}{n_{l}}\sum_{i=1}^{n_{l}}{||IP(l(i))||^{2}}\right)$$

where *γ*_*l*_ is the bandwidth of kernel, *n*_*l*_ is the number of the lncRNA.

### Calculation of interaction profiles for new lncRNAs

In the prediction process, if an lncRNA only knows very few diseases associated with it, this lncRNA is difficult to predict accurately. This is a common problem in industry, such as the difficulty of recommending products to a new user. This problem is also known as cold-start problem. There are two ways to solve it. The first way is to consider additional information in the definition of node similarity. The other one is to use prior information, e.g. diseases with many connections are more likely to interact with a new unknown lncRNA. We had considered using additional data such as lncRNA sequence information to measure similarity between lncRNAs. But on the one hand, the length of lncRNA sequence is very long(> 300 bp), and it is difficult to find an appropriate algorithm to measure their similarity. On the other hand, it is difficult for some new lncRNA to collect their sequence information. So in this paper, we mainly deal with the cold start problem through the second ways. We will describe this process in detail. First, we calculate the interaction profile for a new lncRNA using the mean of its neighbors’ interaction profile. Taken lncRNA *l*(*i*) as an example, the neighbors of lncRNA *l*(*i*) should be satisfied with the following formula:

$$||IP(l(j)) - IP(l(i))||^{2} \geq \frac{\sum\limits_{k=1}^{n_{l}}{||IP(l(k)) - IP(l(i))||^{2}}}{n_{l}} $$

Here, *n*_*l*_ is the number of lncRNA. In another words, if similarity between *l*(*i*) and *l*(*j*) were larger than the mean of the similarity, *l*(*j*) can be defined as the neighbors of *l*(*i*). *IP*(*l*(*i*)) was the mean of its neighbors’ interaction profile.

$$IP(l(i)) = \frac{\sum\limits_{k\in N({lnc}_{i})}{IP(l(k))}}{|N({lnc}_{i})|} $$

Here *N*(*lnc*_*i*_) is the set of the neighbors of lncRNA *l*(*i*) and |*N*(*lnc*_*i*_)| is the size of *N*(*lnc*_*i*_). Notice that our approach here is different from the traditional approach to dealing with cold-start problem. Typically, the traditional method uses the mean of other lncRNAs interaction profile to fill in the new LncRNA. This is actually based on the popularity to make prediction. In contrast, BiwalkLDA uses local topological structure to predict missing interactions. Given a new lncRNA, we first find all its similar (or nearest) lncRNAs, which are likely to share common disease interactors with our node of interest. So, the key point is the definition of similarity function. Unlike all other algorithms, we assume that these lncRNAs sparsely connected to diseases would contribute more to the given node. It means they are likely to share common disease nodes. For example, an inactive user didnŠt buy Harry Potter, although the book is one of the best seller. How likely does a new user would choose to buy the book. In our model, new users would more likely to learn from inactive users.

### The algorithm of Bi-random walk

Based on the construction of lncRNA similarity network and disease similarity network, we use the bi-random walk algorithm to predict potential lncRNA-disease associations. First, I will explain the idea of bi-random walk algorithm. BiwalkLDA mainly make prediction base on the assumption that similar diseases or lncRNA have similar connection patterns. For example, if we know that lncRNA (i) is associated with disease (j) and lncRNA (i) is very similar to lncRNA (j), it is obvious that we can infer that lncRNA (j) may also associate with disease (j). So far we have constructed disease similarity networks and lncRNA similarity networks. Bi-random walk algorithm actually constructed a linear model based on similarity. Suppose we want to predict the relationship between lncRNA (i) and disease (j).

$$a_{ij} = \sum_{k=1}^{n_{l}} a_{i,k}*{sim}_{d}(k,j)$$

Here *a*_*ij*_ represents the possibility that lncRNA(i) and disease(j) are related. *sim*_*d*_(*k, j*) represents the similarity of disease(k) and disease(j). So the process of calculation is actually to traverse every disease k and add *a*_*i*,*k*_∗*sim*_*d*_(*k*,*j*) up. It can be seen as a linear model based on similarity. Considering that we want to keep part of the original *a*_*ij*_, the formula can written as below:

$$a_{ij} = (1-\beta)*a_{ij} + \beta*\sum_{k=1}^{n_{l}} a_{i,k}*{sim}_{d}(k,j)$$

Note that we need to normalize the similarity to ensure that *a*_*ij*_ is always less than 1. The above formula is based on disease similarity to make predictions. Similarly, we can make predictions based on the similarity of lncRNA and then combine the two results together to make final prediction. So the whole process of the algorithm can be divided into three steps: (1)First, we predict new scores based on disease similarity and lncRNA similarity according to random walk algorithm. (2)Then, we use the mean of two scores as the result of this round of prediction. (3)The two steps are repeatedly performed until maximum number of iterations. Let’s go into the details of the algorithm. We do row normalization on both lncRNA similarity network and disease similarity. This is because random walk is actually a linear prediction model based on similarity. The similarity should be normalized so that the prediction results are between 0 and 1.

$$S_{d}(i,j) = S_{d}(i,j)/\sqrt{D_{S_{d}}(i, i)*D_{S_{d}}(j, j)}$$.

Here $D_{S_{d}}(i, i)$ is the sum of the *i*^*th*^ row of *S*_*d*_. Similarly, we normalized the similarity of lncRNA as following formula:

$$S_{l}(i,j) = S_{GKL}(i,j)/\sqrt{D_{S_{GKL}}(i, i)*D_{S_{GKL}}(j, j)}$$

Here $D_{S_{GKL}}(i, i)$ is the sum of the *i*^*th*^ row of *S*_*GKL*_. Adjacent matrix A also needs to be initialized. Scores of all known lncRNA-disease association are set to 1/n where n is th total number of known lncRNA and disease associations. Scores of Other unobserved associations are set to zero.

$$S_{ini}^{0} = \frac{A}{sum(A)}$$

Here *S*_*ini*_ represent the initial probability and the sum of initial probabilities is 1. Because the importance of predicting results based on different similarity networks may be different. We introduce two parameter l and r as the numbers of maximal iterations in the left and right random walks on these two networks. The more iterations, the more important the prediction through this similarity network is. The iterative process can be described by the following formula:

$$R_{d} = \beta S_{ini}^{t-1}*S_{d} + (1-\beta)S_{ini}^{0} $$

$$R_{l} = \beta S_{l}*S_{ini}^{t-1} + (1-\beta)S_{ini}^{0} $$

$$S_{ini}^{t} = \frac{R_{d}+R_{l}}{2} $$

Here *S*_*d*_, *S*_*l*_ represent disease and lncRNA similarity networks. *S*_*ini*_ represents initial score of all disease-lncRNA association. *β* is the decay factor which control the degree of retention of initial information. *R*_*l*_ represents the score of random walk on the lncRNA similarity network and *R*_*d*_ represents the score of random walk on the disease similarity network. In the iterative function, we use the averaged value of *R*_*d*_ and *R*_*l*_ as $S_{ini}^{t}$ in step t. This process can be seen as a combination of lncRNA similarity and disease similarity to make predictions. When the number of iterations reached max(l,r), $S_{int}^{t}$ is the final result which represents the possibilities of all lncRNA-disease association. The pseudocode of bi-random walk algorithm can be seen in Algorithm 1. 
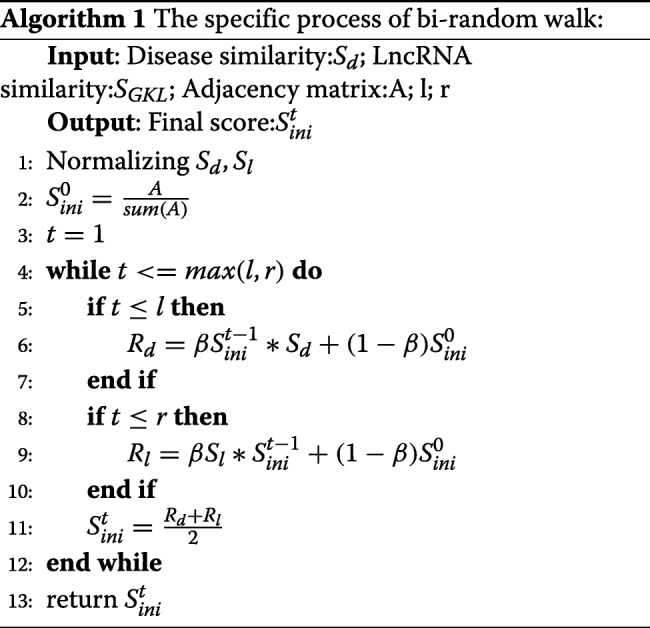


## Data and materials

Known lncRNA and disease associations were downloaded from the LncRNADisease database [17], which is commonly used as the gold standard in predicting lncRNA-disease associations question. In the experiment, we used three databases which are downloaded from three different versions of 2012, 2014 and 2015 (Table 1). in 2012, experiments confirm there exists 276 interactions between 112 lncRNAs and 150 diseases. In 2014, this number has increased to 319 interactions between 131 lncRNAs and 169 diseases. In 2015, it suggests that there are 621 interactions between 285 lncRNAs and 226 diseases.

**Table 1:** Detailed information for three datasets

Datasets	Version	No. of lncRNA	No. of disease	No. of interaction
Dataset1	2012	112	150	276
Dataset2	2014	131	169	319
Dataset3	2015	285	226	621

## Results

We use leave-one-out cross validation (LOOCV) to test the performance of BiwalkLDA. LOOCV is a widely-used strategy to evaluate the quality of the algorithms. In each turn, one known association was set as a test sample. All other lncRNA-disease association were set to training set to train model. All associations that are not observed will be considered as a candidate set and will be scored by BiwalkLDA. A correspond rankList can be generated based on the predicted results. Then true positive rates (TPR, sensitivity) and false positive rates (FPR, 1-specificity) can be calculated by giving different thresholds. Based on the calculated values of TPR and FPR, the receiver-operating characteristics (ROC) curves can be plotted. Then we use the areas under ROC curve (AUC) as evaluation criteria of algorithmic performance which reflects the global prediction accuracy in different situation. The value of AUC closed to one means a perfect prediction, while the AUC value of 0.5 indicates purely random performance.

### The effects of parameters

#### The effects of *α*

**Fig. 1:**
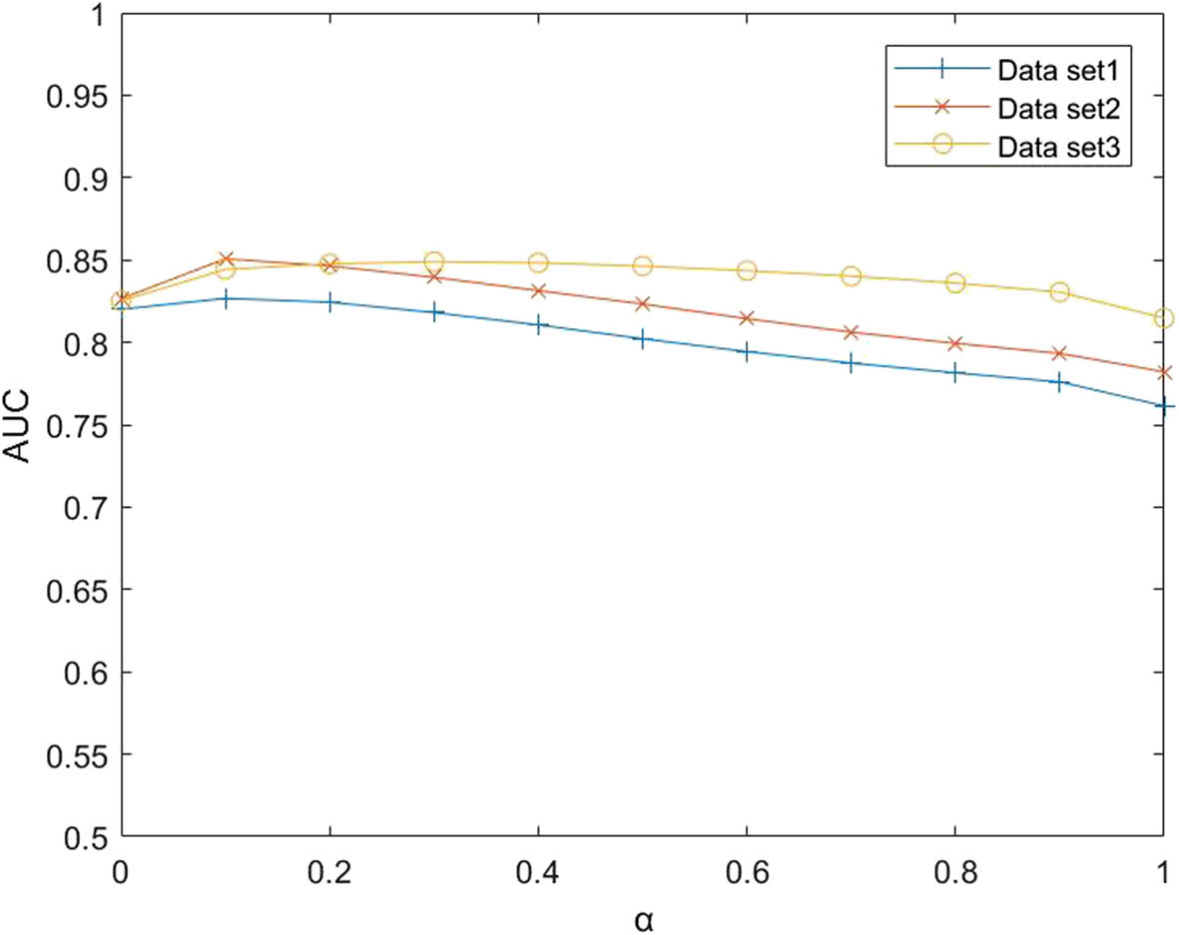
The effect of parameters *α* on three different data sets

In the section of disease similarity, we use a linear model to fuse *S*_*GO*_ and *S*_*GKD*_. Here *α* is a hyperparameter that control the proportion of *S*_*GO*_ and *S*_*GKD*_. If *α* = 1, disease similarity only be calculated base on gene ontology information. If *α* = 0, disease similarity only be calculated base on known disease-lncRNA associations. BiWalkLDA use gene ontology information as a supplement to *S*_*GKD*_, which makes the generalization ability of the algorithm stronger. To test the performance of the algorithm under different *α* values, we changed *α* from 0 to 1 and increased 0.1 per time. Then we use BiwalkLDA to make prediction. The experimental results are shown in Fig. 1, When *α*=0.1, BiwalkLDA obtain the best results on dataset1 and dataset2. On dataset3, it reaches the peak when *α*=0.3. It can see that small changes in *α* do not have much impact on the results. Therefore, we recommend the region of *α* could be set between 0.1 and 0.3 for using BiwalkLDA. The experimental results show that the fusion of *S*_*GKD*_ and *S*_*GO*_ can improve the accuracy of the algorithm. Meanwhile, the algorithm can achieve good performance even if we only use the GO similarity network. It indicates that the algorithm still works in the absence of disease-lncRNA association information.

#### The effects of *β*

**Fig. 2:**
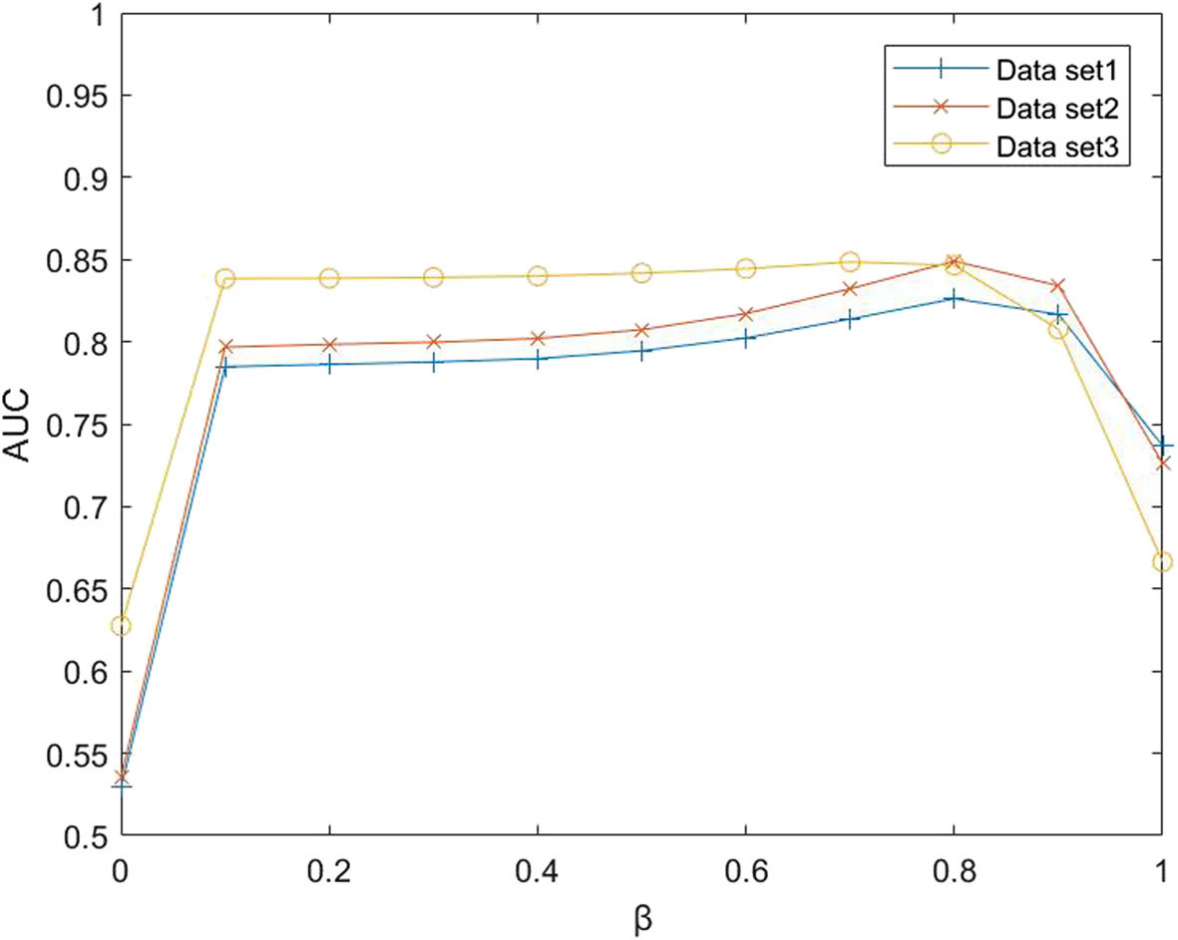
The effect of parameters *β* on three different data sets

*β* is a decay factor in bi-random walk algorithm. *β* determines the degree of retention of initial information in each iteration. if *β*=0, all initial information will be retained. If *β*=1, all initial information will be used to predict new score in each turn. Obviously, it will result in a poor performance if *β* is either 0 or 1 are inappropriate and will result in a poor performance. To test the performance of the algorithm under different *β* values, we increased *β* rom 0 to 1 in 10 steps, and run BiwalkLDA. The value of *β* was changed from 0 to 1 and increased 0.1 each time and then using BiwalkLDA to make prediction. The experimental results are shown in Fig. 2. When 0.1 ≤*β*≤0.9, the results of the algorithm varied slightly. It indicts that BiWalkLDA is robust to *β*. BiWalkLDA performs the best AUC when *β*=0.8 in dataset1 and dataset2 and performs the best AUC when *β*=0.7 in dataset3. Intuitively, if the initial data is sufficient, a smaller *β* is more appropriate. Because dataset3 contains more known lncRNA-disease associations, the optimal *β* in dataset3 is less than the other dataset. Finally, we set *β* = 0.8 as default in three datasets.

#### The effects of l and r

In bi-random walk algorithm, the parameters l and r are used to limit the number of random walk steps in the disease and lncRNA similarity network respectively. l and r can actually be understood as the importance of disease similarity and lncRNA similarity. If the value of l is larger, it means that we will predict more based on disease similarity. Values of different l and r make the algorithm applicable to different data. If l or r equals 0, the algorithm actually degenerates into a single random walk algorithm. This means that we only make predictions through lncRNA similarity or disease similarity alone instead of combining them. To test the performance of the algorithm under different l and r values. We grid search the combination of l and r in a certain range. The value of l and r were increased from 1 to 7 and increased 1 each time. Then we use BiwalkLDA to make prediction and calculate the AUC values by LOOCV. The experimental results are shown in Table 2. The experimental results show that when the values of l and r are relatively close, BiwalkLDA perform well. This shows that prediction through lncRNA similarity and disease similarity are equally important. Finally we set l=6 and r=6 as default in three dataset.

**Table 2:** The effects of parameters l and r in dataset1

	r = 1	r = 2	r = 3	r= 4	r = 5	r = 6	r=7
l = 1	0.7618	0.7230	0.6902	0.6714	0.6585	0.6448	0.6304
l = 2	0.8124	0.7890	0.7292	0.6985	0.6802	0.6702	0.6564
l = 3	0.8008	0.8214	0.8140	0.7295	0.7010	0.6838	0.6713
l = 4	0.7919	0.8092	0.8230	0.8243	0.7285	0.7000	0.6850
l = 5	0.7848	0.7989	0.8115	0.8238	0.8267	0.7269	0.6988
l = 6	0.7778	0.7911	0.8006	0.8119	0.8236	0.8268	0.7255
l = 7	0.7729	0.7834	0.7920	0.8007	0.8116	0.8233	0.8263

### Comparison with other algorithms

**Fig. 3:**
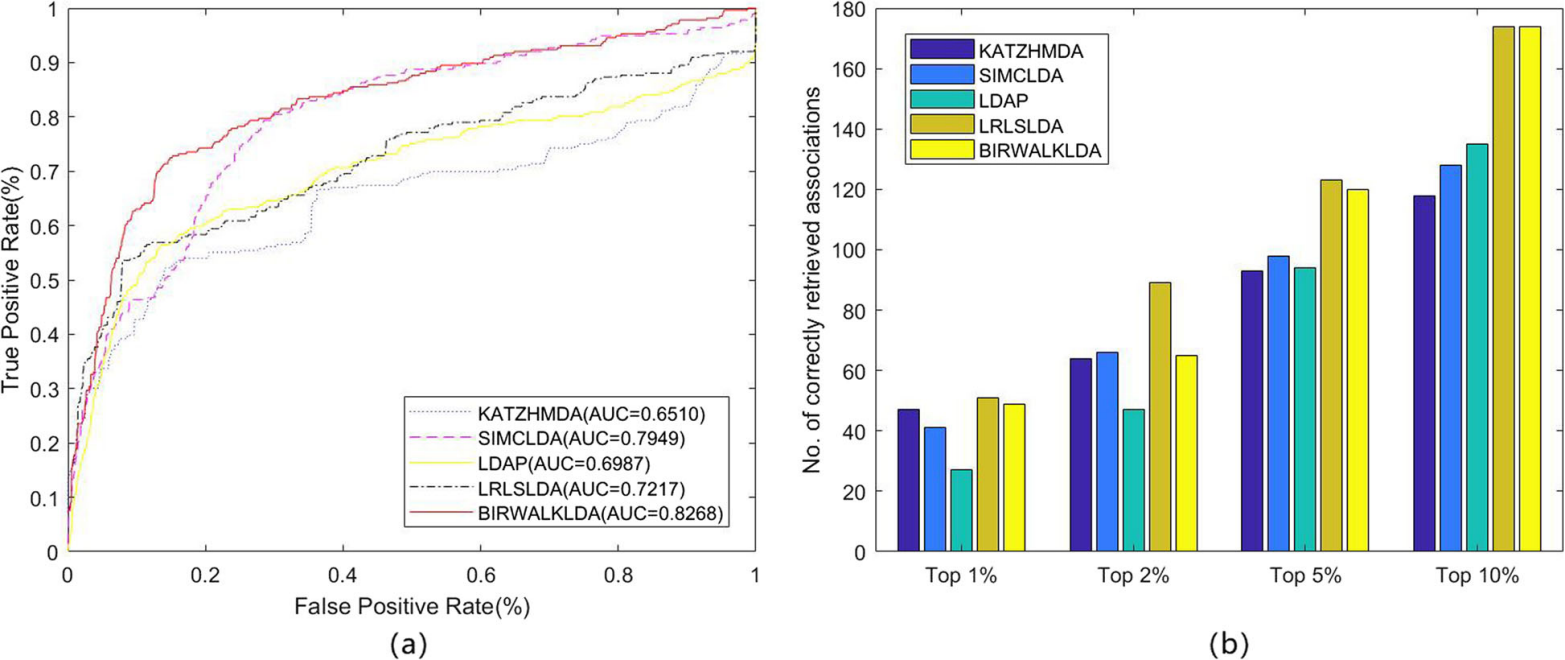
Comparison of predicting methods on dataset1. **a** Receiver operating characteristic curve of all algorithm using LOOCV (**b**) Number of correctly retrieved known lncRNA-disease association for given percentage

**Fig. 4:**
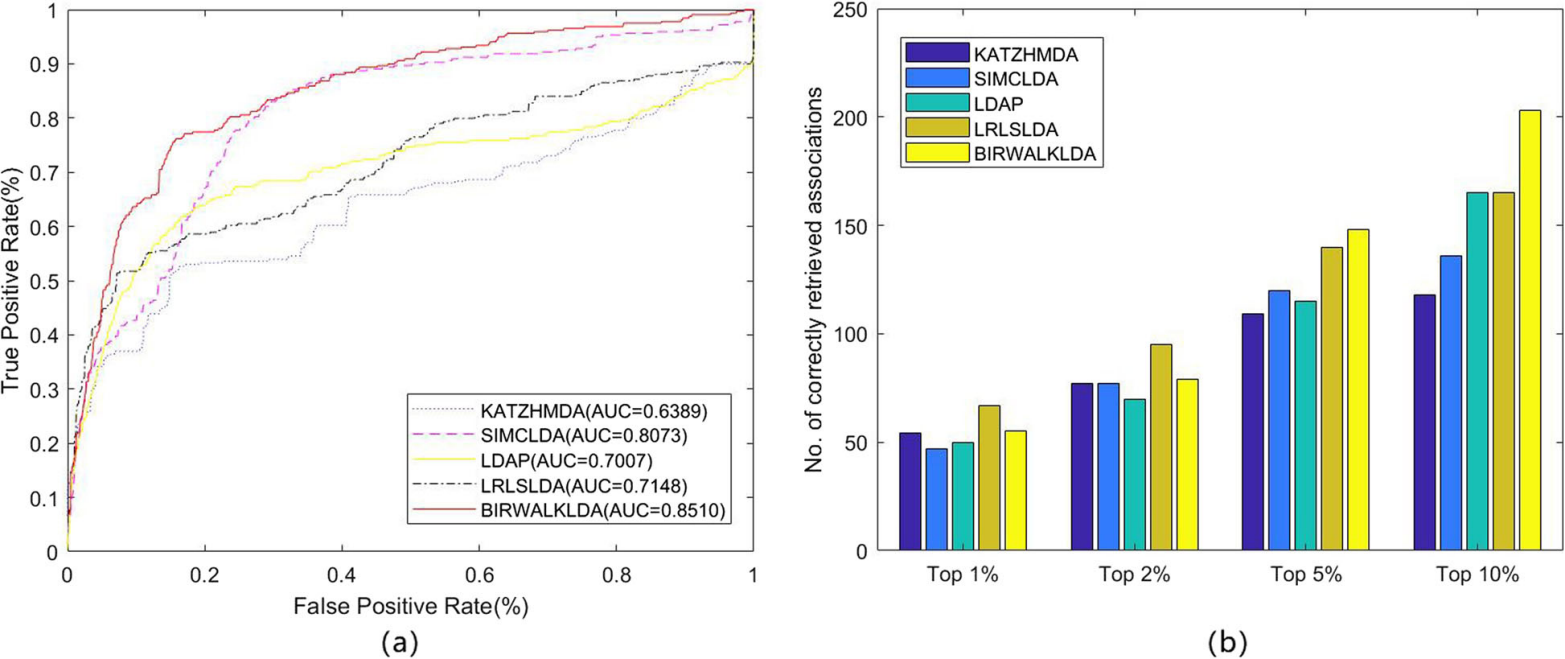
Comparison of predicting methods on dataset2. **a** Receiver operating characteristic curve of all algorithm using LOOCV (**b**) Number of correctly retrieved known lncRNA-disease association for given percentage

**Fig. 5:**
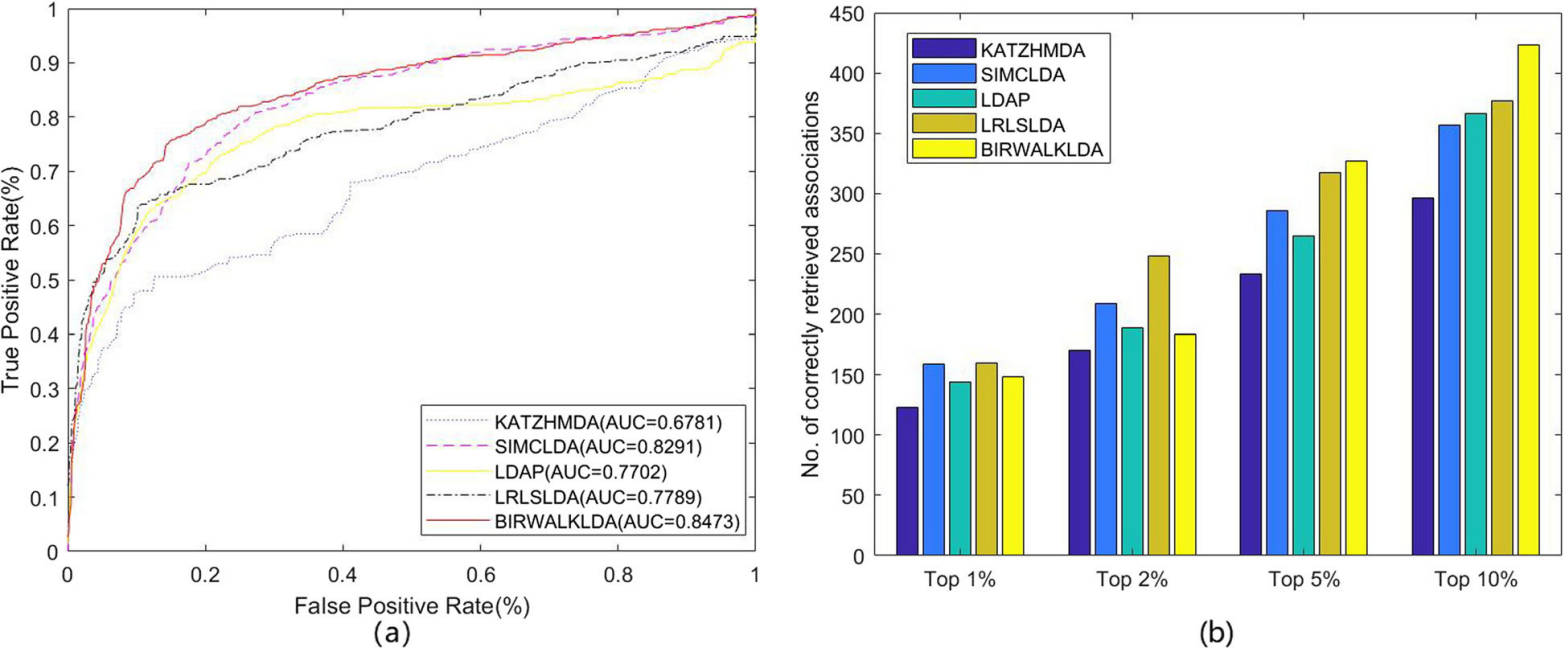
Comparison of predicting methods on dataset3. **a** Receiver operating characteristic curve of all algorithm using LOOCV (**b**) Number of correctly retrieved known lncRNA-disease association for given percentage

To test the performance of the BiwalkLDA, we compared BiWalkLDA with three the-state-of-art computational methods (LDAP, LRLSLDA, SIMCLDA) of lncRNA-disease association prediction in three datasets. The results of the algorithm are measured by AUC value and number of correctly retrieved association. Because limited code can be used, we also compare our algorithm with KATZHMDA which is be used to predict disease-microbe association. LRLSLDA used Laplacian normalization operation and construct cost function in lncRNA and disease space. Then making prediction by minimize the cost function to obtain optimal classifier [15]. LDAP fused different data source and make prediction based on bagging SVM classifier [16]. SIMCLDA predicted lncRNA-disease association based on inductive matrix completion [12]. KATZHMDA integrated known microbe-disease associations and gaussian interaction profile kernel similarity for microbes and diseases and make prediction based on katz algorithm [21]. On dataset1, we can see that BiwalkLDA obtained an AUC of 0.8268 which is higher than others others(LRLSLDA:AUC=0.7217, KATZHMDA:AUC=0.6510, LDAP:AUC=0.6987, SIMCLDA:AUC=0.7949) as shown in Fig. 3a. In addition to AUC, we also use the numbers of correctly retrieved association to measure the performance of the algorithm. If a predicted association in the first percent k of the candidate set, this association will be regard as a correctly retrieved association under given threshold k. So the numbers of correctly retrieved association can reflect the accuracy of the algorithm in top k% and AUC reflects the global performance of the algorithm. The experimental results are shown in Fig 3b. BiWalkLDA can predict more correctly retrieved association in Top10%. But it can also be seen that LRLSLDA performs better at lower thresholds in term of the numbers of correctly retrieved association. This result actually indicates that BiwalkLDA is more inclined to make global optimal predictions. This phenomenon can be explained as follows: (1)BiwalLDA processing samples with less information separately may significantly increase the AUC value, but it may also make incorrect predictions. (2)More comprehensive sequencing results can be obtained based on lncRNA similarity network and disease similarity network. This means that only samples that meet both of these inference criteria will be given a higher ranking. However, if one sample conforms to the similarity inference of lncRNA but does not conform to the other, it will not be given a high ranking. This problem can be solved by using non-linear algorithm. The results on the other two datasets are similar, so we will not discuss them one by one (Figs. 4 and 5). It can be concluded that BiWalkLDA also achieve the best result(AUC 0.8510 in dataset2 and AUC 0.8473 in dataset3) and BiwalkLDA is robust enough to different parameter selection.

### De novo lncRNA-disease prediction

**Fig. 6:**
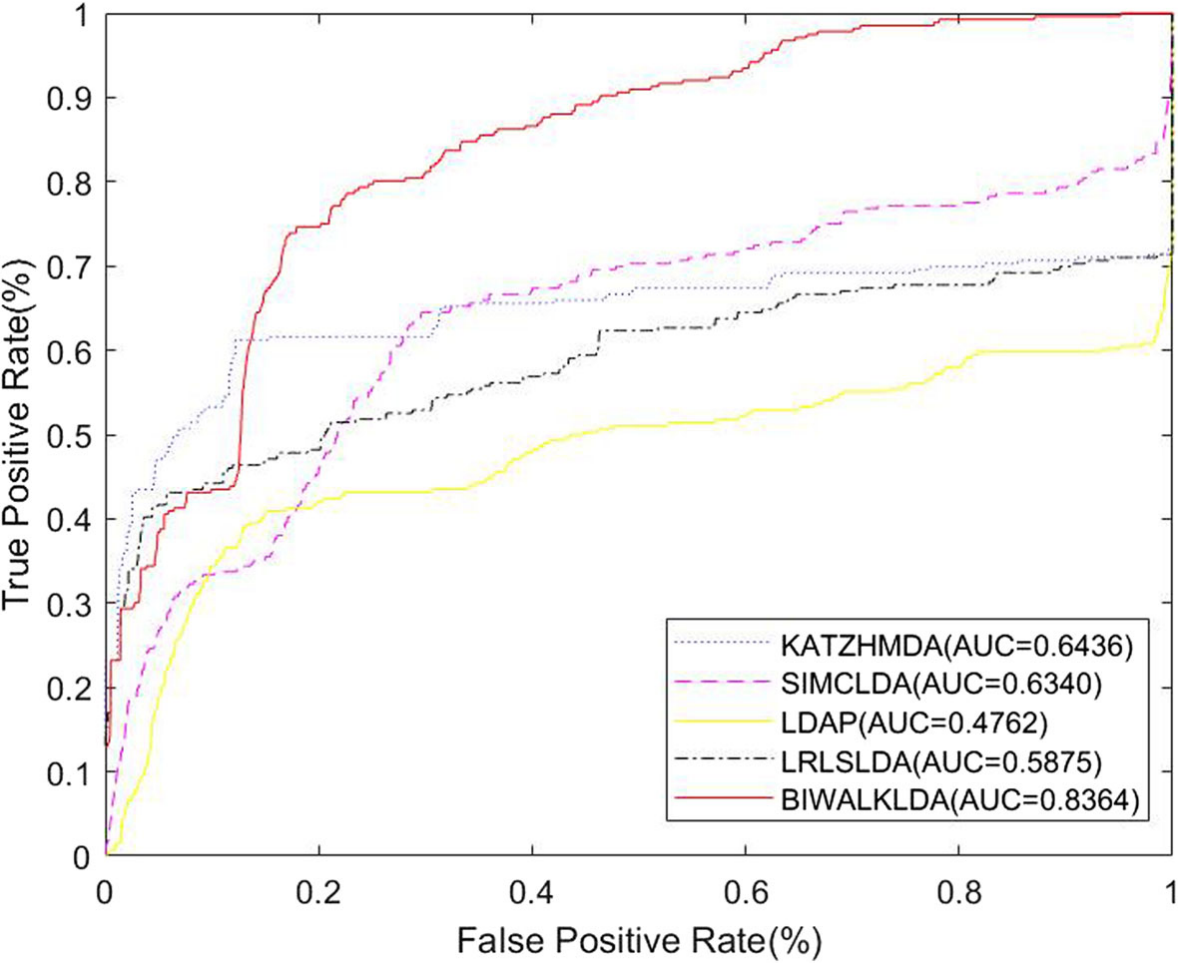
Comparison of predicting methods in de novo prediction test on dataset1

In section of disease similarity, we combine gauss similarity and gene ontology similarity. Fusion of multiple similarities network not only improves the performance of the algorithm, but also strengthen generalization ability of BiwalkLDA. To assess the performance of BiWalkLDA, we conduct de novo lncRNA-disease association prediction in dataset1. In the process of de novo prediction, each queried disease d(i) would be removed all known lncRNA-disease association of this disease. Different computational methods were used in the prediction problem. Notice that we still know the gene ontology information of the disease. The experimental results are shown in Fig 6. The performance of BiWalkLDA only has a slight drop(AUC:0.8364) and is much higher than other algorithms. The result shows that BiWalkLDA can make good prediction even if there is absence in disease-lncRNA association information and combining heterogeneous data sources can deal with data missing situation. Note that AUC of LDAP only has 0.4762. This result is lower than random guess which AUC value is 0.5. This is because we are actually testing the performance of the algorithm in the absence of data. LDAP treats this problem as a classification problem and using a bagging SVM classifier to make prediction. If there is a serious lack of data, the features learned will be inaccurate and the effect of classification will be poor. There are two reasons for the good performance of BiwalkLDA. 1) Gene ontology information was used as a supplementary data. 2) BiwalkLDA used the neighborhood information to predict connections for new unknown lncRNAs.

### Case studies

We conduct case study for prostate cancer to test the performance of BiWalkLDA in predicting new lncRNA for a certain disease. Prostate cancer (PC) is the most commonly diagnosed malignancy and the third leading cause of cancer death among men in developed countries. Predicting prostate cancer related lncRNA will help us to understand the mechanism of prostate cancer and provide a high probability set of candidate lncRNA for scientist. We use dataset3 to make prediction and confirm the result by searching related paper. Prediction results of the BiwalkLDA are showed in Table 3. It can see that eight prostate cancer-related lncRNA(H19, MALAT1, HOTAIR, MEG3, PVT1, GAS5, NEAT1, UCA1) in the top ten candidates have been confirmed by previous studies. Long non-coding RNA H19 and H19-derived microRNA-675(miR-675) were significantly down-regulated in the metastatic prostate cancer cell line M12 compared with the non-meta-static prostate epithelial cell line P69 [22]. MALAT1 was up-regulated in human prostate cancer tissues and cell line [23]. HOTAIR as an androgen-repressed lncRNA is markedly up-regulated following androgen deprivation therapies and in castration-resistant prostate cancer [4]. MEG3 decreased significantly in prostate cancer tissues relative to adjacent normal tissues [24]. Region surrounding rs378854 which is identified as a novel function prostate cancer-specific genetic variant interacts with the MYC and PVT1 promoters [25]. GAS5 promotes the apoptosis of prostate cell, and exonic sequence, i.e. GAS5 lncRNA, is sufficient to mediate this activity [26]. Nuclear enriched abundant transcript 1 (NEAT1) was identified as the most significantly over-expressed lncRNA in prostate cancer by using a combination of chromatin immunoprecipitation (ChIP) and RNA-sequencing data [27]. UCA1 was abnormally up-regulated in tumor tissues from prostate cancer patients and patients with high UCA1 levels had a significantly poorer prognosis [28]. Successful predictions for prostate cancer prove that BiWalkLDA can help us to find new relationships between lncRNA and disease base on historical data.

**Table 3:** Top ten reported lncRNAs for prostate cancer

Rank	Name of lncRNA	PMID
1	H19	PMID: 24988946
2	CDKN2B-AS1	Unconfirmed
3	MALAT1	PMID: 23845456
4	HOTAIR	PMID: 26411689
5	MEG3	PMID: 26610246
6	PVT1	PMID: 21814516
7	BCYRN1	Unconfirmed
8	GAS5	PMID: 23676682
9	NEAT1	PMID: 25415230
10	UCA1	PMID: 26550172

## Conclusion

Many recent studies suggest that lncRNAs are strongly associated with various complex human diseases and they play important roles in the gene expression regulation and post-transcription modification. Predicting lncRNA-disease association can help understand the biological mechanism of disease and reduce the cost of experimental verification. However, discovering the relationship between lncRNA and disease by means of computational model is still a very challenging problem. Therefore, the development of computational tools is much in demand. Although many computational models have been proposed. Their prediction accuracy still has a lot of room to improve. To improve the performance of existing algorithms, we present a novel algorithm, BiwalkLDA based on bi-random walks for the prediction of lncRNA-disease associations. It integrates gene ontology and interaction profile data together to calculate disease similarity, to solve the cold-start problem by using the local structure of lncRNAs neighbors information. Four the-state-of-art computational methods and BiwalkLDA are applied to predict lncRNA-disease associations on three different datasets. Results show that BiwalkLDA is superior to every other existing algorithms in terms of both accuracy and recall. There are still many problems to be dealt with. Existing models are based on small-scale datasets. Although algorithms can achieve high accuracy, their results are often repetitive. If the dataset is too large, the existing algorithms can not be applied to large-scale data. In future work, we will consider to develop more effective algorithm to solve this problem.

## Data Availability

The datasets used and/or analysed during the current study are available from the corresponding author on reasonable request.

## Abbreviations

AR: Androgen
IMC: Inductive matrix completion
LOOCV: Leave-one-out cross validation
ROC: receiver-operating characteristics
